# Developing Models of Disease Transmission: Insights from Ecological Studies of Insects and Their Baculoviruses

**DOI:** 10.1371/journal.ppat.1003372

**Published:** 2013-06-13

**Authors:** Bret D. Elderd

**Affiliations:** Department of Biological Sciences, Louisiana State University, Baton Rouge, Louisiana, United States of America; Duke University Medical Center, United States of America

Mathematical models of disease outbreaks play a crucial role in guiding public health policy [1]–[3] and have provided critical insights into the ecological dynamics of pathogen-regulated populations [4], [5]. Remarkably, the models used to describe epidemics among human populations have been quite successful at describing epizootics in many animal populations, including forest- and crop-defoliating insect pests [6] whose population dynamics are often driven by baculoviruses [7]. Given the variety of insect species affected by baculoviruses and their ubiquity in nature, these viruses may help in the development of disease models that not only increase our understanding of disease transmission in general, but directly lead to better agricultural and forestry management through environmentally benign pest control.

## What Are Baculoviruses?

From a medical and microbiology perspective, baculoviruses have been developed as expression vectors and for research tools in medicine such as vaccine development [8], [9]. Interesting examples of each occur throughout the literature. For instance, under stressful conditions often brought on by disease, a host's eukaryotic translation initiation factor 2α (eIF2α) phosphorylates to limit protein synthesis. To counter this defense, baculoviruses can encode a truncated protein kinase that disrupts eIF2α and increases translational activity for virus production [10]. In the realm of vaccine development, insertion of subunits of the targeted disease (e.g., arboviruses like dengue fever) into a baculovirus genome allows for rapid production in insect cell lines. These subunits can then be extracted and used in vaccines [11]. However, to most virologists and microbiologists, the ecology of these viruses may be less well-known.

**Figure 1:**
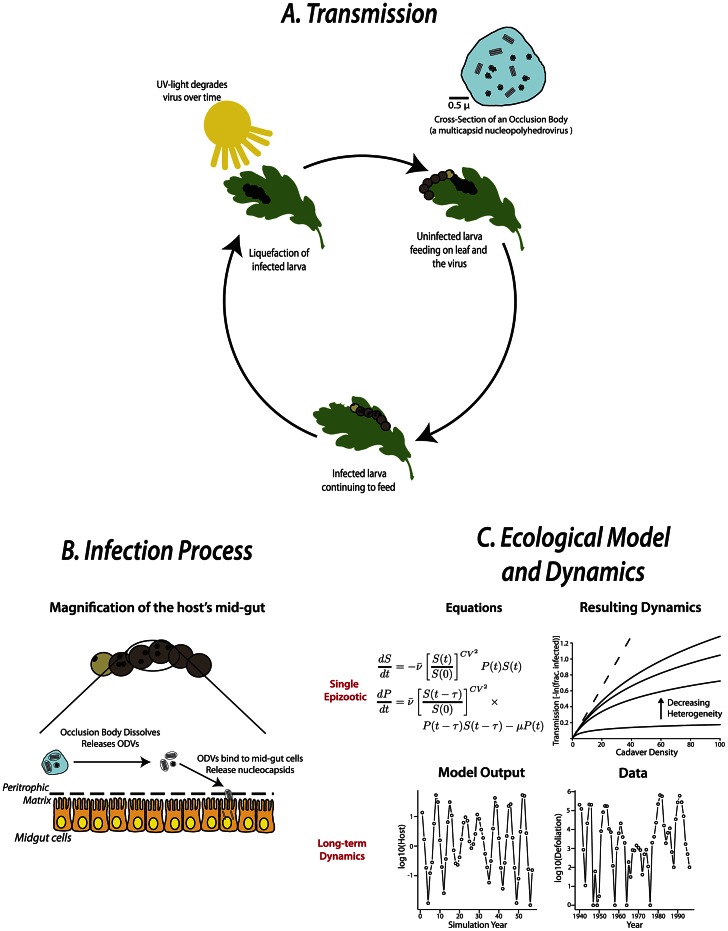
Typical baculovirus transmission cycle, the infection process, and resulting ecological dynamics in lepidopteran larvae. A) The transmission cycle begins when an uninfected larva consumes occlusion bodies (OBs) containing either multiple or single copies of the baculovirus. If enough OBs are consumed, the virus establishes in the midgut of the host and eventually spreads throughout the body of the now infected larva. Eventually, liquefaction of the host occurs as the host's body tissue is consumed by the virus, resulting in millions of OBs within a single larva. The virus triggers the larval integument to split open, releasing the newly formed OBs into the environment to continue the cycle. Over time, the OBs degrade due to ultraviolet light exposure. B) Within the larvae, the OB's protein coat dissolves, releasing the occlusion-derived viruses (ODVs). The ODVs bind to the midgut cells and, in turn, release their nucleocapsids into the cells' cytoplasm. The nucleocapsid uncoats and begins reproduction once it is transported to the nucleus. C) During a single epizootic, the change over time (t) in the density of susceptible individuals (S) and pathogen-infected cadavers (P) in the environment depends upon the mean transmission rate (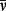) between the two, the coefficient of variation (CV) associated with that rate, the time between infection and death (τ), and the degradation of the virus in the environment (μ). For a given transmission rate 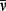, a population's heterogeneity has a dramatic impact on disease transmission. As individuals become more similar, heterogeneity between them decreases and overall transmission increases. When all individuals share the same transmission rate, transmission becomes linear (the dotted line in the “Resulting Dynamics” figure) as pathogen-infected cadaver density increases. A long-term dynamic model for the gypsy moth derived from the equations shown [7] does a reasonably good job of replicating the pattern of forest acres defoliated in Maine from 1940 to 1996.

Baculoviruses, as they occur in nature, consist of double-stranded circular genomes of viral DNA enveloped within a double membrane. These occlusion-derived viruses (ODV) contain one or multiple nucleocapsids with each capsid holding a single viral genome. One or more ODV, in turn, are “occluded” within a protein matrix, referred to as occlusion bodies, that protect the virus from the environment with the proviso that the occlusion bodies eventually degrade under UV light [8], [12]. An individual insect becomes infected during the larval stage when it consumes leaf tissue on which the virus resides (Figure 1A). Once consumed, the occlusion body dissolves and the ODV are released within the midgut. ODV bind and fuse with midgut cells, releasing the nucleocapsids into the cytoplasm (Figure 1B). The nucleocapsids are transported to the nucleus, uncoat, and begin replication, producing budded virus particles [13]. In most baculovirus infections, the budded virus then spreads throughout the larva via the tracheal system and hemocytes. Late in infection, the host's tissues are filled with virions that are occluded in millions of occlusion bodies, which are released upon death when the host liquefies [8]. This horizontal-transmission cycle continues when uninfected larvae consume the recently released occlusion bodies. The virus, depending upon the host, may also be transmitted vertically between mother and offspring [12], although the relative importance of vertical transmission remains unclear.

## What Determines Baculovirus Transmission Dynamics?

The above question has plagued researchers since the first recorded incidence of baculovirus transmission appeared in ancient China, when the disease infected cultivated silkworms. In the nineteenth century, the definitive cause of baculovirus infections was identified when occlusion bodies were first seen under a light microscope [13]. Of the numerous species affected by baculoviruses, the forest-defoliating gypsy moth (*Lymantria dispar*), which causes widespread ecological and economic damage in Eastern North America, has received considerable attention. Therefore, much is known about what governs its transmission dynamics and how these dynamics depend upon the host, the pathogen, and even the leaf tissue that is consumed with the virus.

As is true in other animal populations, gypsy moth larvae vary in their degree of disease resistance. Individual levels of resistance are heritable and linked to a negative trade-off with fecundity such that the least resistant individuals produce the most offspring [5]. At low densities, selection favors these highly reproductive individuals. During an epizootic, however, increased reproduction comes with a price, as the most fecund but least resistant individuals are selected against. Over the long-term, these relatively rapid changes in evolutionary selection pressure due to the negative trade-off between host resistance and reproduction help drive the boom-and-bust cycles observed in natural populations [5].

While evolution plays an important role in virus-driven epizootics, the ecological community in which the interaction between the host and its pathogen occurs can also dictate the frequency and intensity of epizootics. For instance, numerous plant species, such as oak trees, produce secondary compounds when eaten by insect herbivores that affect baculovirus infection rates [14], which can change the timing and intensity of an epizootic [15]. Additionally, predators play an important role in controlling the insect. Depredation of larvae and pupae by predators keeps populations at lower densities than would be expected if the virus alone determined the boom-and-bust cycles [7], [16]. Interestingly, one of these predators, the white-footed mouse (*Peromyscus leucopus*), whose populations are enhanced by feeding on gypsy moth pupae, has been linked to increased incidence of Lyme disease due to its own boom-and-bust cycles [17]. In general, baculovirus transmission dynamics can be driven by both evolutionary and ecological processes, which may also be prevalent in other host-pathogen systems.

While a great deal of research has focused on the host, considerable attention has also been paid to the baculovirus pathogen. For instance, the deletion of a single gene, the *egt* gene within the gypsy moth baculovirus, causes the host to alter its normal behavior of climbing into the tree canopy to die [18]. The decrease in climbing height results in a decrease in virus transmission among susceptible larvae. This represents a clear example in which pathogen genes change host behavior. Examples of changes in host behavior have been observed in numerous other pathogen systems [8], [18], but few studies have found such a clear link to the pathogen genome.

While much can be gained from studying various aspects of the host and the pathogen, baculovirus systems can lend important insight into coevolutionary dynamics between the two. Across the range of the host, considerable genetic variation occurs such that transmission dynamics change based on the origin of the host and the virus, leading to differences in infection rates [19]. This suggests that host resistance and pathogen virulence can both play a role in determining the population cycles seen in nature. Since the pathogen and the host may both be under selection pressure, these systems are ripe for exploring coevolutionary dynamics.

## What Are the Advantages of Using Baculovirus Systems for Studying Disease?

Succinctly, both the host and the pathogen in baculovirus systems can be manipulated easily and infection easily diagnosed under a light microscope. This means that multiple replicates of a single experiment can be carried out in either the laboratory or, maybe more importantly, in nature. This would be unheard of in systems where vertebrates serve as the host (e.g., bluetongue in ruminants). In turn, these experiments can be designed to address specific hypotheses about transmission dynamics or to develop mathematical models of disease transmission [5], [20], [21]. The models based on both biological mechanisms and experimental data can be used to predict single epizootics or long-term population dynamics (Figure 1C). The output from these models, which are similar in form to those used to describe endemic diseases in human populations, can be directly compared to long-term ecological data. If the new model fits the data well (e.g., the evolution of host resistance must be invoked to properly describe the boom-and-bust cycles seen in the data), then the mechanism used in constructing the model is important for describing past population dynamics and for determining the timing of future outbreaks. If this newly formulated model does not fit the empirical data, then the mechanism is not important for describing disease transmission and can be left out of future models. In this way, ecological models of disease transmission continue to be developed and refined as hypotheses are tested and data collected. For gypsy moth populations, this methodology has been used to show the effects of both pathogen and predator in controlling host populations [7], the evolutionary trade-off between host resistance and fecundity [5], forest composition [15], [16], and environmental degradation of the pathogen [21] on epizootic dynamics.

## What Can Baculovirus Transmission Teach Us about Diseases of Human Concern?

Baculovirus systems have similar attributes to a number of diseases of human concern such as herpesvirus and poxvirus, which are also large-encapsulated DNA viruses. For example, many viruses like cholera and endemic flu depend upon the transmission of infectious particles released into the environment [22], [23] as do baculoviruses. Understanding the importance of a pathogen reservoir in determining epidemic intensity is in many ways similar to baculovirus reservoirs that initiate epizootic outbreaks in insect populations. If the virus degrades rapidly in the environment, as has been demonstrated for the gypsy moth's baculovirus, the epizootic will come to an abrupt end rather than slowly fade out as many disease models assume [21]. The similarity in transmission dynamics does not end there. For instance, models developed to describe the importance of heterogeneity among individuals in determining epizootic dynamics in gypsy moth populations (Figure 1C) arose from ideas originating with HIV research, where heterogeneity in transmission can vary due to the number of sexual partners [3]. Most models previously developed to describe disease transmission assumed that every individual had similar transmission rates.

Baculovirus systems can also provide us with a richer understanding of how climate change may affect disease transmission. Concern over whether global warming will increase epidemic frequency of diseases such as cholera and malaria has provoked considerable debate in the ecological disease literature [24], [25]. When considering how environmental change may affect disease transmission among humans, empirical data is often missing [26]. While it is also true that empirical data associated with nonhuman hosts during epizootics is also lacking, it can be readily collected. This is particularly true for baculovirus systems where insects serve as hosts. By manipulating temperature or precipitation, for example, in experimental plots over the course of an epizootic, insight into how environmental change affects disease transmission and which mechanisms may be responsible for the changes observed can be easily gained.

## How Can Baculoviruses Decrease Pesticide Use?

Control of insect pests of crops and forests has historically depended on the use of synthetic pesticides [27]. Bioinsecticides have emerged as a potential alternative to their chemical cousins, especially given the rise in organic agriculture and due to environmental concern [28]. In general, bioinsecticides vary in their effectiveness and host specificity. Some bioinsecticides are able to kill a broad range of insects, such as the common bioinsecticide *Bacillus thuringiensis* (Bt) [29]. However, broad range chemical or biological insecticides may be undesirable because beneficial insects may also be affected. Baculoviruses hold promise as a viable potential alternative since they have much narrower host ranges and many are species specific [12]. This comes with an important caveat: to date, little attention has been paid to evolution of host resistance or virus virulence when developing baculoviruses for biocontrol. The rapid decrease in virulence of myxoma virus in controlling introduced European rabbit (*Oryctolagus cuniculus*) populations in Australia stands out as a cautionary tale of how evolutionary responses of the host and the pathogen render the virus essentially ineffective [30]. In general, the development of baculoviruses as an effective bioinsecticide or a biological control agent has met with mixed success [31], [32]. Yet, given their potential, the development of these viruses for biological control is an active area of research that could lead to better management practices in both agriculture and forestry.

## Conclusion

These relatively common viruses have been the focus of research efforts for over a century. This research has led to a deeper understanding of the causes behind some of the more dramatic population cycles in nature and has shown that pathogens can indeed control the populations of their hosts [4]. Research into the mechanisms driving host-pathogen dynamics has resulted in the development of disease transmission models with broad applicability [6]. Additionally, these viruses may even lead to reduced dependence on pesticides and enhanced agricultural production methods and may be a viable alternative to pesticide dependence [8]. Further research is needed, however, to translate breakthroughs in basic science into applications.
